# Advertising Online Surveys on Social Media: How Your Advertisements Affect Your Study

**DOI:** 10.1093/poq/nfaf018

**Published:** 2025-06-16

**Authors:** Anja Neundorf, Aykut Öztürk

**Affiliations:** Professor, School of Social & Political Sciences, University of Glasgow, Glasgow, UK; Lecturer, School of Social & Political Sciences, University of Glasgow, Glasgow, UK

## Abstract

Although the recruitment of online survey participants through paid social media advertisements is becoming increasingly common among survey researchers, we know little about how the content of advertisements influences the recruitment process. Our study systematically compares the effects of several approaches—being vague or explicit about the survey theme and offering material incentives—relying on 23 advertisements conducted in Turkey and Spain between May 2021 and June 2022, recruiting more than 30,000 respondents. Our article documents the important trade-offs that the content of an advertisement creates regarding cost and sample composition. We find that incentive-based advertisements can produce samples much closer to national population benchmarks; however, this also depends on the type of incentive. Thematic advertisements, which mention the political content of a survey, consistently return the cheapest samples, yet certain groups are overrepresented in these samples. Incentive-based advertisements also produce a generally higher response quality. We conclude our article by providing practical advice on which kind of advertisement to use, discussing the generalizability of our findings to other countries, and listing the main limitations of our study.

## Introduction

Using social media platforms to recruit survey participants is becoming increasingly common among political scientists. There are a few reasons behind this. First, paid advertisements on social media produce affordable samples, without necessarily leading to a decline in response quality. For example, Neundorf and Öztürk (2023, p. 13) report costs as low as £0.19 and £0.77 per successful survey completion, across studies conducted in Turkey, Spain, the Czech Republic, and the United Kingdom (UK). Second, recruitment through social media occurs through a more transparent and direct process, compared to working with a commercial survey company. This allows the researcher to have direct control over the recruitment process and to know where survey responses are coming from.

Most importantly, especially for comparative political scientists, social media advertisements facilitate geographical coverage that is not otherwise available. While high-quality and reliable commercial online panels are unavailable in most countries, Meta, which owns Facebook and Instagram, offers direct access to a regular user base of three billion people worldwide. In recent years, political scientists have relied on paid social media advertisements to conduct public opinion research in countries like Brazil (Boas, Hidalgo, and Toral 2021), Egypt (Williamson and Malik 2021), Germany (Reinermann et al. 2023), Ghana (Erlich, Soehl, and Chen 2023), Indonesia (Ananda and Bol 2021), Kenya and Tanzania (Rosenzweig and Zhou 2021), Korea and Japan (Kim and Boas 2020), Turkey (Elçi 2022; Öztürk 2023), and Tunisia (Finkel, Neundorf, and Ramírez 2024). Our research team conducted complex survey experiments with participants from more than thirty countries, all recruited through social media advertisements (see Neundorf et al. 2023, 2024).^1^

The challenge of recruitment through social media is that it is the researcher’s task to convince social media users to take an online survey. Social media companies, such as Meta or TikTok, never guarantee a certain number of survey participants to researchers; they only sell advertising space. Thus, the researcher has to create an advertisement that will draw the attention of social media users and convince them to take an online survey.

Which images and texts should researchers use in their advertisements? While there is a large literature in medical sciences demonstrating that advertisement content affects who is recruited into medical studies (see, for example, Choi et al. 2017; Machado et al. 2019; Ryan et al. 2019; Grow et al. 2020), there is a gap in the political and social science literature on this topic (Zindel 2023, p. 34). The primary concern among political scientists has so far been evaluating whether recruitment through social media is a viable alternative to other recruitment methods (Jäger 2017; Sances 2018b; Boas, Christenson, and Glick 2020; Rosenzweig et al. 2025; Zhang et al. 2020). Taking the research a step further, our article systematically compares the impact of different types of advertisements. To our knowledge, this is the first article undertaking this effort from a political science perspective. As such, this article significantly contributes to the social science literature on recruitment through social media and the broader academic literature on the effects of content choices used in paid social media advertisements.

The rest of our article is composed of five sections. In the next section, we explain the advertising structure on Meta platforms and describe our four categories of advertisement content: neutral, thematic, incentive based, and mixed. After that, we introduce the design of the three studies we conducted. In the results section, we discuss the effects of advertisement content on costs, sample composition, and response quality. We summarize our findings in the fifth section of our article. Finally, we conclude by discussing the shortcomings of our research and the avenues for future research.

## Advertisement Strategies on Meta for Public Opinion Research

### Advertising on Meta

Meta owns the social media platforms that are most frequently used by researchers to recruit research participants, that is, Facebook and Instagram (Zindel 2023). While using these platforms, researchers need to buy advertisement space from Meta and create their advertisements.^2^ Meta advertisements consist of an image or video uploaded by the advertiser and some text entered above and/or below the image. Advertisers also need to choose the advertisement budget, the optimization algorithms that Meta will use to deliver the advertisement, and the audience of the advertisement.

Meta allows advertisers to limit the audience of their advertisements to certain demographic groups through a feature called targeting. This tool can be used to create samples that are closer to population benchmarks at the expense of some increase in cost (Zhang et al. 2020; Neundorf and Öztürk 2023). Finally, researchers can link their advertisements to surveys hosted on Qualtrics or any other survey platform. After the advertiser finalizes the advertisement, Meta’s machine-learning algorithms conduct a review of the content to determine if there are any violations of Meta’s advertising principles. Once the review process is complete, advertisements are published on Facebook and Instagram, and Meta users clicking on these advertisements are directed to the survey page linked to the advertisement.

### Advertisement Content Strategies

We distinguish four types of advertisement content strategies that political scientists usually adopt: neutral, thematic, incentive based, and mixed.

First, scholars often create advertisements that do not reveal the topic of their survey and do not offer any material incentives. We call this base category *neutral advertisements*. Using neutral messages is recommended for minimizing the sample bias (Zhang et al. 2020, p. 563). Following this concern, it is a common practice to create advertisements that draw attention only to the practice of taking a survey. Images such as questionnaires or pencils can be used to create neutral advertisements (see, for example, Boas, Christenson, and Glick 2020).

Second, many researchers are explicit about the topic of their surveys when recruiting study participants via social media. They can do this to ensure that their advertisement reaches the right audience, or they may simply want to draw the attention of social media users with a more interesting advertisement. It is argued that “carefully worded advertisement texts which inform the participant about the goals of a study” can motivate potential participants to join the survey and that these advertisements can perform better than incentives (Pötzschke and Braun 2017; Tuten, Bosnjak, and Bandilla 1999, p. 637). We call these advertisements, which reveal the content of the survey, *thematic advertisements*. For example, one of Williamson and Malik’s (2021) advertisements combines images of Egyptian riot police with texts such as *“Violent or Just? What is happening in Egypt? We want to hear from you! - University Researchers.”*  Sances (2018b) uses texts asking US-based social media users if they are supporting the Democratic Party.

The use of incentives is another common strategy for recruitment in online studies. MTurk and many commercial panels rely on material incentives provided to all participants in a survey. Since the payment of respondents over social media is not straightforward, researchers using paid social media advertisements generally use raffles. The most commonly used prizes in these raffles are iPads and other Apple products (Samuels and Zucco Jr. 2014; Avenburg 2019) and vouchers from websites such as Amazon (Sances 2018a; Munger et al. 2020) or Netflix (Finkel, Neundorf, and Ramırez 2024). There are also examples of cash payments to research participants (Kim and Boas 2020; Boas, Hidalgo, and Toral 2021).

While there has been significant research on the use of incentives in traditional surveys, the literature for online studies is much more limited, and it offers contradictory findings (Brosnan, Kemperman, and Dolnicar 2019, p. 4). This means that there are many unanswered questions in this research context. First, do incentive-based advertisement campaigns on social media cost less or more than other advertisement campaigns, when the cost of the incentive is also considered (i.e., what is the total cost per completed survey for incentive-based advertisement campaigns versus those that do not use incentives)? Second, how does the use of incentives affect the sample composition? Finally, does using incentives lead to low response quality since people motivated only through incentives would not provide attentive answers?

What if a researcher chooses to use both incentives and thematic references in the advertisement content? We call these advertisements “mixed advertisements.” Our study also explores whether combining incentives and thematic appeals means realizing the best of both worlds.

The four advertisement content strategies discussed so far are shown in table 1, depending on whether they offer incentives and whether they refer to the survey content.

**Table 1.** Advertisement content strategies.

		Reference to survey content	
		No	Yes
Material	No	Neutral	Thematic
Incentive	Yes	Incentive based	Mixed

Advertisements falling under one of these four categories can vary within each other as well. For example, incentive-based advertisements can vary based on the type of incentive (e.g., vouchers versus in-kind goods), chances of winning (e.g., 1 in 2 versus 1 in 500), the value of the prize (e.g., $25 versus $500), and the expected value of the raffle, which equals the multiplication of the chance of winning and the value of the prize. We study the effects of these variations, too, in our article.

## Research Design

This article presents results from three separate studies conducted as part of broader projects with a substantive focus on support for democracy. These three studies are summarized in table 2.

**Table 2.** A summary of studies and advertisements.

Study No	Date	Country	Advertisement strategies	Number of advertisements
1	May 9–20, 2021	Turkey	Incentive based	3
			Neutral	4
			Thematic	2
			Mixed	3
2	Feb. 19–Mar. 7 2022	Spain	Incentive based	2
			Neutral	1
			Thematic	1
3	June 10–23, 2022	Turkey	Incentive based	5
			Neutral	1
			Thematic	1

All three studies rely on the systematic and controlled comparison of advertisement content. We published a series of advertisements with different content choices for each study. Across each of them, however, all advertisements had the same settings concerning targeting tools, optimization algorithms, the duration and date of advertisements, and daily budget.^3^ Thus, we gave all advertisements an equal chance to draw social media users' attention, making them comparable to each other for our purposes. While our studies cannot be called experimental, as social media users were not randomly assigned to one of our advertisement campaigns, they can be called a “controlled comparison.” We also registered the data collection and analyses before any data collection commenced.^4^

### Study 1

Study 1 was conducted in Turkey. We believe there are good reasons to choose Turkey as one of the cases in such a study. Turkey represents countries in which paid social media advertisements are especially promising for recruiting survey participants. Turkey has a large population and high internet penetration; as a result, more than 40 million Turkish citizens use Facebook regularly. It is a middle-income country; hence it is cheaper to advertise and deliver material incentives in Turkey than in advanced economies. Also, like in many other non-Western countries, it is hard to find commercial panel companies in Turkey that will provide high-quality and representative online panels.

On the other hand, Turkey is currently ruled by an electoral authoritarian regime (Laebens and Öztürk 2021). The government imposes limitations on the freedom of expression and the freedom of internet in the country. Given that most of the people living in the world now live under this regime type (Wiebrecht et al. 2023), it is important to use such a case in our studies.

**Figure 1.**
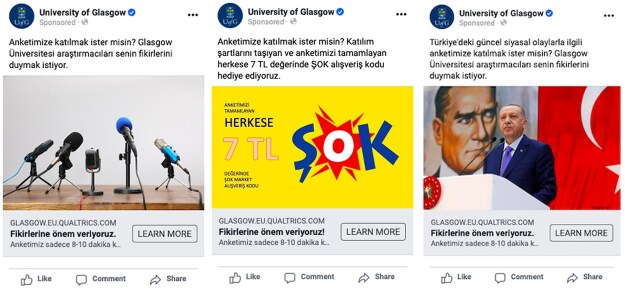
Examples of neutral, incentive-based, and thematic advertisements in Study 1 (from left to right). All advertisements used in our studies are also presented in the Supplementary Material.

Our primary goal in Study 1 was to compare incentive-based, neutral, thematic, and mixed advertisements in terms of their effects on costs, sample composition, and response quality. We also studied the effect of variations within these categories on these outcomes. As presented in table 2, we published twelve different advertisements for Study 1, each falling under one of the four main advertisement strategies but also representing some variation from other examples of the same advertisement strategy. Some examples of these advertisements can be seen in figure 1; we also document all advertisements in the Supplementary Material.

We published three incentive-based advertisements that all offered the same expected gain.^5^ Two of these advertisements featured grocery store vouchers, while the third incentive-based advertisement offered participation in a raffle for an Apple iPad.

Four of our advertisements in Study 1 belonged to the neutral category. The baseline neutral advertisement combined a vague text with an image of microphones. The text read, *“Do you want to participate in our survey? University of Glasgow researchers want to hear your opinions.”*^6^ In the second (third) neutral advertisement, we used the image of a man (woman) combined with a neutral text. In the fourth neutral advertisement, we used human images matching the gender of the targeted social media user.

Study 1 further included two thematic advertisements. The first thematic advertisement, which can also be seen in figure 1, used both a political image and a political text. This advertisement showed a photo of the Turkish president, Recep Tayyip Erdogan, in front of a Turkish flag and an Ataturk poster. The political text accompanying this image was *“Do you want to participate in our survey on current political issues in Turkey? University of Glasgow researchers want to hear your opinions.”* In the second variation of thematic advertisements, we used political text together with an image of microphones. Our goal was to explore how the two components of a Facebook advertisement—the image and the text—fared vis-à-vis each other in drawing the user’s attention.

Finally, Study 1 also included three mixed advertisements that combined the thematic text, mentioning the political nature of the survey, with the promise of material incentives. The image used in these advertisements was the same as in incentive-based advertisements.

### Study 2

Study 2 was conducted in Spain. Like Turkey, Spain has a large social-media user base, approaching 40 million people. Yet, unlike Turkey, it is a high-income country. Furthermore, unlike Turkey, Spain has strong democratic institutions. Thus, studying social media advertisements in Spain allowed us to explore the extent to which our findings from Turkey traveled to countries with more advanced economies and democratic institutions.

We used a simple design in Study 2, comparing only four advertisements to each other. The neutral advertisement mimicked the baseline neutral advertisement in Turkey, which combined the image of a microphone with a vague text, as seen in figure 1. For the thematic advertisement category, we used an advertisement that included both political text and a political image. Unlike the political image in Study 1, however, in Study 2, we used the image of a political rally.^7^ We used two incentive-based advertisements in Spain, which both offered “vouchers,” but did not specify the brand of vouchers. These advertisements offered the same expected gain as the advertisements in Turkey, and they only varied concerning the chances of winning and the value of the prize. We did not use any mixed advertisements in Spain.

### Study 3

Finally, building on the results of the first two studies, we published seven advertisements in Study 3. This study was also conducted in Turkey. The main purpose of Study 3 was to compare the performance of incentive-based advertisements with each other. We designed five incentive-based advertisements with varying chances of winning, expected gain, and brand use. In addition to incentive-based advertisements, we also published one thematic and one neutral advertisement to test whether our previous findings would be replicated. In the former, we combined an image of a rally full of Turkish flags with a political text. In the latter, we used the same image of microphones, as seen in figure 1.

### Targeting and Settings

While we used both political and demographic targeting tools in the first two studies, we could only use demographic targeting tools in the third study, due to Meta’s withdrawal of the political targeting feature in the meantime.^8^ The third study also included less strict targeting of educational categories. We used conversion-based optimization algorithms in all our studies.

### Number of Observations

Following our registration plan, we kept all advertisements open until they recruited at least 250 participants. Power calculations demonstrate that 250 respondents per sample are enough to detect a difference of 15 percent in gender distribution and the proportion of university-educated individuals. Also, Meta’s advertisement delivery usually stabilizes after the first fifty recruits, making it possible to generalize based on these samples.

Once we reached 250 participants per advertisement, we closed the worst-performing advertisements and continued data collection for our substantive project with the remaining advertisements. When presenting the results regarding costs, we limit our samples to the first 250 participants for each advertisement. This is because costs tend to increase over time. When we present results about sample composition and response quality, we use our entire sample from each study. Sample composition and response quality depend less on the timing of recruitment.

## Results

We present the results in the following four subsections. The first three subsections compare the four advertisement strategies, grouping variations of these strategies. The last subsection focuses on variations within incentive-based advertisements.

### Recruitment Cost Across Advertisement Strategies

Table 3 summarizes the total costs across the advertisement strategies and three studies. The total cost equals the amount paid to Meta to buy advertisement space and the amount spent on incentives, if any, per successfully recruited respondent.^9^ For each advertisement strategy, we present the performance of the least costly advertisement, the costliest advertisement, and the average performance of all advertisements. The results regarding the least costly advertisement show the potential of each strategy, while the average cost and the cost of the most expensive advertisement inform us about the extent to which the performance of that strategy can vary.

**Table 3.** Comparison of costs across studies.

Study 1: Turkey		
Advertisement category	Advertisement	Average cost
Incentive based (Three ad variations)	Cheapest of three variations	$0.62
	All advertisements	$1.01
	Most expensive of three variations	$1.72
Neutral (Four ad variations)	Cheapest of four variations	$1.25
	All advertisements	$1.53
	Most expensive of four variations	$1.69
Thematic (Two ad variations)	Cheapest of two variations	$0.37
	All advertisements	$0.71
	Most expensive of two variations	$1.04
Mixed (Three ad variation)	Cheapest of three variations	$0.69
	All advertisements	$1.16
	Most expensive of three variations	$2.10

Study 2: Spain		
Advertisement category	Advertisement	Average cost
Incentive based (Two ad variations)	Cheapest of two variations	$1.89
	All advertisements	$1.92
	Most expensive of two variations	$1.95
Neutral	Only ad of its category	$0.76
Thematic	Only ad of its category	$0.16

Study 3: Turkey		
Advertisement category	Advertisement	Average cost
Incentive based (Five ad variations)	Cheapest of five variations	$0.34
	All advertisements	$0.52
	Most expensive of five variations	$0.85
Neutral	Only ad of its category	$0.39
Thematic	Only ad of its category	$0.10

*Note*: Results are based on the first 250 recruits through each advertisement. In all studies, we used targeting based on age, gender, and education. In Study 1, we also used targeting based on political characteristics and we especially aimed to recruit lower-educated people. This explains the price difference between Study 1 and Study 3. For this reason, the analysis should focus on comparing price differences within studies rather than across studies.

The most striking finding from table 3 is that thematic advertisements are consistently superior to other advertisement strategies in terms of cost. Using thematic advertisements, we could recruit participants at half the cost compared to other advertisements. Surprisingly, the overall costs of most of the incentive-based advertisements were less than the neutral advertisements, even when we consider the cost of incentives. However, the difference between the cheapest and most expensive advertisements also demonstrates that the performance of these ads can vary significantly. We discuss these variations in more detail below.

The performance of the mixed advertisements used in Study 1 was very close to the performance of incentive-based advertisements featuring the same incentive, but slightly worse. This may be because our mixed advertisements used the same image as an incentive-based advertisement but with a different text. These results suggest that the advertisement image matters more than the text. Based on these results, we chose not to publish mixed advertisements in later studies.

### Sample Composition

Table 4 shows the sample composition across different advertisement strategies, based on the responses provided by our survey participants.^10^ In terms of demographic characteristics, table 4 lists the proportion of females, young people (aged 18–34), old people (aged 55 or higher), and people with a university degree. In terms of political characteristics, the table shows the proportion of respondents who were very interested in politics and respondents who showed strong identification with a political party.^11^ To better interpret sample compositions, we provide a population benchmark, based on weighted averages from nationally representative surveys.

**Table 4.** Demographic and political compositions of samples depending on advertisement.

Study 1: Turkey						
Advertisement strategy	Demographic characteristics				Political characteristics	
	Female (%)	Young (%)	Old (%)	Graduate (%)	Very interest. (%)	Strong partisan (%)
Population	51	36	24	18	14	25
Incentive based						
(n = 5,347)	51	50	19	15	14	49
Neutral						
(n = 5,433)	40	24	35	19	35	59
Thematic						
(n = 2,441)	26	35	29	24	49	62
Mixed						
(n = 4,378)	49	50	17	17	18	51


Study 2: Spain						
Advertisement strategy	Demographic characteristics				Political characteristics	
	Female (%)	Young (%)	Old (%)	Graduate (%)	Very interest. (%)	Strong partisan (%)
Population	51	22	39	26	13	n/a
Incentive based						
(n = 669)	60	27	32	40	18	41
Neutral						
(n = 296)	44	13	60	60	42	45
Thematic						
(n = 3,578)	36	28	39	49	45	57
						

Study 3: Turkey						
Advertisement strategy	Demographic characteristics				Political characteristics	
	Female (%)	Young (%)	Old (%)	Graduate (%)	Very interest. (%)	Strong partisan (%)
Population	51	36	24	18	14	25
Incentive based						
(n = 17,347)	50	41	21	31	19	25
Neutral						
(n = 386)	28	17	45	55	50	39
Thematic						
(n = 2,536)	23	20	39	44	54	47

*Note*: Population benchmarks are based on the Comparative Study of Electoral Systems (CSES) survey Wave 5 in Turkey and European Social Survey data (ESS) Round 9 in Spain. We weighted population estimates with *pspweight* variable for the ESS survey and *E1010 2* variable for the CSES survey. In all studies, we used targeting based on age, gender, and education. In Study 1, we also used targeting based on political characteristics, and we especially aimed to recruit lower-educated people. This explains the differences between Study 1 and Study 3 with respect to education levels and political characteristics.

An important lesson from table 4 is the superiority of incentive-based advertisements over thematic advertisements in creating samples closer to national population benchmarks. The difference between samples is clearest when comparing the political characteristics of the samples. Incentive-based advertisements produce samples that are not very different from the general population in terms of their interest in politics. On the other hand, half of the people recruited through thematic advertisements profess to be very interested in politics. This is three times more than the general population of both Spain and Turkey. People recruited through thematic advertisements also tend to be more partisan than other participants.

There are important differences between thematic and incentive-based advertisements concerning demographic results as well. Compared to the general population, samples recruited through thematic advertisements are formed of a significantly higher number of men, older people, and university graduates. On the contrary, incentive-based advertisements are successful in appealing to female social media users and users without a university degree.

Samples recruited through neutral advertisements usually fall between these two extremes. However, it is important to note that neutral advertisements led to especially disproportionate samples in the third study in Turkey, where the cost of the advertisements was relatively lower. This shows that neutral advertisements produce cheaper samples at the expense of sample composition. In other words, to the extent that these advertisements are successful, they are successful at drawing the attention of politically interested and university-educated social media users.

### Response Quality

We have four outcome variables to measure response quality: passing an attention check, responding to an open-ended question, the number of words used to respond to an open-ended question, and filling out a follow-up survey one month after the original survey. A full list of survey questions is included in Supplementary Material section 2.

We would expect the advertisement content to have some indirect effects on response quality due to its effects on sample composition. To isolate these confounding effects, we conducted multiple regression analyses, in which we control for demographic and political variables introduced in the previous section. We also added study-fixed effects to all our models. We only included respondents completing the entire survey in our analyses to ensure that we did not confuse the behavior of skipping the response quality questions with the behavior of leaving the survey. Sample sizes are 11,081 (for the attention check question), 12,208 (for models about the open-ended question), and 8,924 (for the recontact success). We did not attempt to recontact respondents in Study 2; this is why the sample size is smaller for this question.^12^

**Figure 2.**
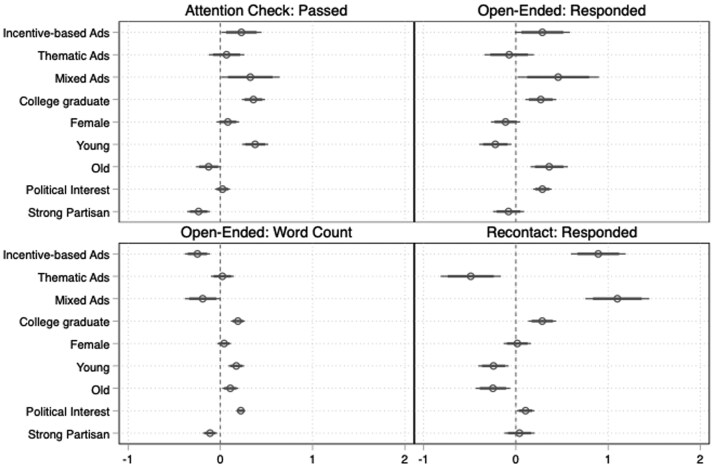
The effect of advertisement content on response quality.

We used logit models for binary outcome variables (attentiveness, responding to the open-ended question, recontact success) and OLS for the continuous outcome variable (the length of response to the open-ended question). Study fixed effects are added to all models. The baseline condition for advertisement strategies is the neutral advertisement strategy. Since our samples are relatively bigger, we present results with 0.95 and 0.99 significance levels. Results are presented in figure 2; we also provide the statistical tables in Supplementary Material section 6.

To begin with, we measured the attentiveness of respondents with an attention-check question, asking respondents to choose the response option “Do not know” as their answer. To capture the straight-lining tendencies, this question was located at the end of a matrix question toward the end of the survey. We considered those who answered “do not know” to this question as having passed the attention check. While 78 percent of respondents recruited through incentive-based advertisements passed our attention check, only 69 percent of the other participants did. The positive relationship between incentive-based advertisements and attentiveness is still significant when we control for confounding variables (*p *= 0.007).

Second, we asked respondents an open-ended question on how they would define “democracy.” Although they had the option to skip the question, 88 percent of our respondents answered this question. While the percentages are very close to each other across advertisement groups, the multiple regression analysis, reported in figure 2, reveals that people recruited through incentive-based advertisements were more likely to respond to an open-ended question compared to participants recruited through neutral advertisements (*p *= 0.013). This is not the case for thematic advertisements.

Third, using Stata commands, we counted how many words our participants used to respond to the open-ended question. The average response, excluding nonresponses, was nine words long, while the median response was six words long. Unsurprisingly, people who had high political interest left longer responses. The median response for respondents recruited through thematic (incentive-based) advertisements, excluding nonresponses, was seven (five) words. Our multiple regression analysis, in which we used the square root of the word count as the outcome variable, also demonstrates that respondents recruited through incentive-based and mixed advertisements left shorter responses (*p *= 0.000), while there is no statistically significant difference between respondents recruited through thematic or neutral advertisements.

Finally, as part of the first and third studies in Turkey, we conducted follow-up surveys. We sent emails and SMS messages to respondents who had left their contact information, asking them to fill out a follow-up survey.^13^ We then created a variable capturing whether the respondent completed the follow-up survey. Participants recruited through incentive-based advertisements were significantly more likely to complete the follow-up surveys, compared to respondents recruited through neutral advertisements (*p *= 0.000).

To sum up, we find some differences concerning response quality, but these differences do not always move in the same direction. The substantially biggest difference is that incentive-based advertisements perform better when we want to recontact survey participants.

### Variations within Incentive-Based Advertisement Strategies

In addition to comparing thematic, neutral, mixed, and incentive-based advertisement strategies, we have also analyzed how variations within incentive-based advertisement strategies affected the recruitment process.^14^ We only present results concerning the cost in this section; results for sample composition are presented in the Supplementary Material.

All our incentive-based advertisements followed the same design template. The text above the advertisement image reads: *“Do you want to participate in our survey? We will give [chances of winning] participants who meet the conditions of participation and complete our surveys a [prize] worth [prize value] [through a raffle].”* The advertisement image included the brand's logo, which was used with the company’s permission, and text repeating the information above the image. In the advertisements that did not mention the brand name, we used the drawing of a gift card or gold money. Under the advertisement image, the text always read, “*We value your opinions! Our survey will only take [survey duration] minutes.”*

As can be seen in table 5, we used three incentive-based advertisements in Study 1, each offering the same expected gain of £0.60 ($0.84 by that day’s exchange rates). Two of them featured grocery store vouchers. One of these advertisements offered vouchers worth £0.60 ($0.84) to all respondents who completed the survey. The other advertisement promised users they would be included in a raffle for a voucher worth £4.20 ($5.90), with the chance of winning being 1 in 7. Finally, our third incentive-based advertisement offered participation in a raffle for an Apple iPad, which was worth around £300. Our advertisement noted that participants would have a chance of 1 in 500 to win this iPad.

**Table 5.** Comparison of costs across incentive-based advertisements.

Study 1: Turkey						
Summary	Incentive type					Cost p. person
	Prize	Brand mentioned?	Prize value	Chances of a win	Expected return	
Baseline lottery	Voucher	Yes	£4.20	1 in 7	£0.60	$0.62
Guaranteed win	Voucher	Yes	£0.60	1 in 1	£0.60	$0.68
Very low chance of winning	iPad	Yes	£300.00	1 in 500	£0.60	$1.72


Study 2: Spain						
Summary	Incentive type					Cost p. person
	Prize	Brand mentioned?	Prize value	Chances of a win	Expected return	
Lower chance of winning & voucher	Voucher	No	£8.40	1 in 14	£0.60	$1.89
Very low chance of winning	Voucher	No	£150	1 in 250	£0.60	$1.95

Study 3: Turkey						
Summary	Incentive type					Cost p. person
	Prize	Brand mentioned?	Prize value	Chances of a win	Expected return	
Smaller expected gains	Voucher	Yes	£2.10	1 in 7	£0.30	$0.34
Baseline lottery	Voucher	Yes	£4.20	1 in 7	£0.60	$0.43
Lower chance of winning	Voucher	Yes	£8.40	1 in 14	£0.60	$0.48
Guaranteed winning	Voucher	Yes	£0.60	1 in 1	£0.60	$0.48
No brand references	Voucher	No	£4.20	1 in 7	£0.60	$0.85

*Note:* Advertisements are listed based on the average cost per survey participant. Prize value and expected value are presented in GBP because these amounts were originally planned in GBP. Final costs are converted from GBP to USD based on the average monthly exchange rate when the study was conducted. For Study 1, May 2021 (£1 GBP being equal to $1.4077). For Study 2, February 2022 (£1 GBP being equal to $1.3533). For Study 3, June 2022 (£1 GBP being equal to $1.2318). Source: https://www.exchangerates.org.uk/GBP-USD-spot-exchange-rates-history-2021.html.

In Study 1, we found very small differences in the effects of two advertisements featuring grocery store vouchers. On the other hand, the advertisement featuring an iPad turned out to be less successful than the other two advertisements in recruiting new participants. This might be because of one of three reasons: (i) the chance of winning was very low in this advertisement (1 in 500), (ii) because our advertisement promising an Apple product was not credible enough for social media users, or (iii) because social media users were not very interested in the product.

In Study 2, we wanted to test the first of these explanations and see whether advertisements with grocery store vouchers would also fare badly if they had a very low chance of winning despite offering the same expected gain. For this reason, we set up two different incentive-based advertisements, both promising the same expected return as the advertisement in Turkey: £0.60 ($0.82) per person. The baseline lottery offered a prize of a gift card worth 10 Euros, with a chance of winning 1 in 14. The advertisement with “the very low chance of winning” offered a prize of a gift card worth 175 Euros, with a chance of winning of 1 in 250. Importantly, none of our advertisements mentioned a brand name for the gift card.

There was not a significant price difference between the two incentive-based advertisements in Spain, suggesting that the chance of winning does not matter a lot if the expected return is the same. We took this as evidence that the failure of incentive-based advertisements featuring an iPad in Study 1 was not about the low chance of winning. On the other hand, both incentive-based advertisements in Spain cost significantly more, reaching nearly $2 per successful completion (also see table 3).

There are two alternative explanations for the relative failure of incentive-based advertisements in Spain. First, the purchasing power of the offered material incentives was lower in Spain than in Turkey, as Spain is a richer country. Second, unlike the advertisements in Turkey, our advertisements in Spain did not mention the brand of the gift cards. To understand to what extent these could explain our failure in Spain, we conducted Study 3.

Study 3 included five incentive-based advertisements. Two of them were the same with the most successful incentive-based advertisements from Study 1. These advertisements offered vouchers with an expected gain of £0.60 ($0.76) and a chance of winning 1 in 7 and 1 in 1. We then created a third advertisement that did not include any brand name, as was the case with advertisements in Spain. Our fourth incentive-based advertisement offered half of the expected gain offered by other advertisements, £0.30 ($0.38), while keeping the chance of winning at 1 in 7. The goal was to test how much the failure of our advertisements in Spain was grounded in the lower expected return of our advertisements in Spain. Finally, we designed an advertisement that offered a chance of winning at 1 in 14 while increasing the prize to £8 ($10.6), hence keeping the expected gain at £0.60 ($0.76). This advertisement offered the same chance of winning as one of the advertisements in Spain. Thus, the goal was to see whether advertisements with lower chances of winning could be successful in Turkey.

The results from Study 3 are especially helpful to understand when incentive-based advertisements work. First, the advertisement that did not mention the brand of the incentive is distinguished from other incentive-based advertisements by its poor performance. This finding suggests that the failure of incentive-based advertisements in Spain was also primarily due to the lack of any mention of the brand in the advertisement content. On the other hand, we did not see any differences based on the chance of winning. This, we believe, gives conclusive evidence that the chance of winning does not have much effect on the outcome.

Finally, the advertisement offering a smaller expected return in Study 3 cost us slightly less than all other incentive-based advertisements. Everything else equal, the recruitment process was slower with this advertisement, as fewer social media users opted in. However, the decrease in incentive payments compensated for the decrease in advertisement performance. This suggests that even incentive-based advertisements with smaller expected gains can work well to recruit survey participants, and researchers need to experiment to find the right expected return to offer participants.

## A Summary of Findings: How to Advertise on Social Media?

What are the best advertisement practices on social media for academic researchers? The answer depends on the purpose of the researcher.

First, our studies consistently demonstrated that samples recruited through thematic advertisements cost much less than others. On the other hand, however, these samples are seriously imbalanced: men, older and highly educated people, strong partisans, and people who are very interested in politics are significantly overrepresented in them. In our experience, using targeting tools offered by Meta more aggressively, such as creating additional thematic advertisements targeting only women or lower-educated people, does not help solve this problem. The source of the problem is the low appeal of thematic advertisements for people who are not very interested in politics, and assigning more advertising budgets to recruit these people will only diminish the cost advantage of thematic advertisements. Thus, the trade-off between cost and sample quality is an essential feature of recruitment through thematic advertisements.

Sample imbalances are not a problem if the goal of the advertiser is to reach a specific population, such as strong partisans (e.g., Jäger 2017), or conduct a study that will not be affected by the demographic and political characteristics of the sample. Most scholars, however, use samples recruited through social media to conduct survey experiments and make inferences about the general population. Although the need to match population benchmarks is generally considered to be lower in experimental studies, it is still not possible to predict the average treatment effect correctly when the sample is overly biased, and these biases interact with heterogeneous treatment effects.

We believe that incentive-based advertisements offer the best combination for scholars in terms of the trade-off between cost and sample diversity. In our studies, samples that are closest to national population benchmarks were produced through incentive-based advertisements. These advertisements were usually not costly as well. However, not all incentive-based advertisements perform well. Incentive-based advertisements that do not include the brand name of the incentive fail to convince people to participate in the survey, probably because they do not evoke credibility among social media users. We also find that advertisements offering vouchers are more effective than advertisements offering iPads. Another point in favor of incentive-based advertisements is that these advertisements outperformed neutral and thematic advertisements in three out of four response quality metrics.

To what extent are our insights generalizable beyond the Turkish and Spanish cases? In May 2023, we started advertisement campaigns for two substantive studies (Neundorf et al. 2023, 2024). We ran English- and Spanish-language advertisements in 32 countries spread across all continents; our goal was to recruit 1,500 complete responses in each of these countries. Building on the findings from the current article, we designed all of our advertisements to be incentive based, with no mentions of the political content of our surveys. That cross-national data collection project verified that incentive-based advertisement designs are feasible in most parts of the world. We present these results in more detail in Supplementary Material section 8.

As a final note in this section, it is important to remember that the effects of the advertisement content can interact with the effects of targeting tools offered by Meta. There is already literature on using these targeting tools to meet population benchmarks at certain key variables (see, for example, Boas, Christenson, and Glick 2020; Zhang et al. 2020; Neundorf and Öztürk 2023). Researchers will get the best results when their advertisement content can augment the power of these targeting tools.

## Concluding Remarks: Limitations and Avenues for Future Research

Before concluding our article, we want to discuss some of its shortcomings and avenues for future research.

First, we acknowledge that there are even more combinations of content choices that we did not have the chance to compare in our studies systematically. For example, there are other types of incentives that our studies did not test for practical reasons, such as offering airtime (Rosenzweig and Zhou 2021) or direct cash payments (Kim and Boas 2020; Boas, Hidalgo, and Toral 2021). Second, we tested the impact of mixed advertisements, which combined thematic appeals with incentives, in a specific way: the advertisement image mentioned incentives, and the advertisement text mentioned the political appeal. Some other researchers use the opposite design; for example, Kim and Boas (2020) and Boas, Hidalgo, and Toral (2021) combine neutral images with text mentioning material incentives. These differences can impact the performance of incentive-based advertisements. Relatedly, it is a limitation of our article that we do not perform statistical tests except in the case of data quality.^15^ A promising avenue for future research is the conduct of statistical analyses on advertisements’ performances by combining data from multiple researchers who used social media advertisements in varying contexts.

Second, scholars working in this field should always remember that “temporal validity” is a primary challenge for any study conducted on social media and internet tools (Munger 2019). Technological developments, attempts at government regulations, competitive economic pressures for social media companies, and changing user habits result in rapid changes in this field and the quick “decay” of findings presented in scientific articles. Our article’s findings are arguably less subject to decay than many other studies conducted on social media tools, as we explore the impact of advertisement content rather than some technological tools that may soon be replaced. Furthermore, the insights in this article rely on the results of a series of advertisement campaigns run between June 2021 and June 2023. The consistency of our results over this long period increases our confidence in them. Still, researchers working in this area should follow the changes in social media advertisement tools closely, as well as social media companies’ policies^16^ and the performance of advertisements.

To deal with the challenges of temporal validity, it is essential to find new ways that can facilitate the quick dissemination of academic knowledge. Munger (2019), for example, suggests quickening the publication process for academic articles published on online tools. As another way to handle this challenge, we have launched a network of researchers using social media tools for research purposes and have organized online webinars, in which researchers using paid advertisements can discuss the performance of their advertisements, the strategies they adopted to navigate Meta policies, and the lessons they learned during the process.^17^ We also use this network to circulate working papers and reports written on the topic. It is essential for researchers using social media tools for participant recruitment to contribute to these types of channels. This will help us adapt to changes in the way these social media tools operate.

## Supplementary Material

nfaf018_Supplementary_Data

## Data Availability

Replication data and documentation are available at https://doi.org/10.7910/DVN/8IOK3U.

## Footnotes

1 We share more information about these projects in Supplementary Material section 8.

2 For a recent guideline on how to publish advertisements on Meta, see Pötzschke et al. (2023).

3 We provide details on these settings below, in the subsection named “Targeting and Settings.”

4 The preregistrations are available at the following addresses: https://osf.io/9fs6e, https://osf.io/pzm2r, and https://osf.io/t3nu5.

5 The expected gain equals the multiplication of the chance of winning and the value of the prize. The two advertisements featuring grocery store vouchers differed in terms of the chance of winning and the value of the prize. We discuss these in more detail in the subsection named “Variations within Incentive-Based Advertisement Strategies.”

6 One may argue that identifying foreign researchers, such as “the University of Glasgow researchers,” as the source behind the advertisements can influence the performance of advertisements. This does not threaten the internal validity of our findings, as all advertisements provided the same source information, but it can limit their external validity. We are unaware of any research that directly tests the effects of international sources, and we believe this is an important area for future research. We thank the editors for pointing this out.

7 Meta has recently started requiring special permission for advertisements that include political images. We discuss these new policies in Supplementary Material section 1.

8 Targeting means the publishing of separate copies of an identical advertisement to be separately delivered to demographic groups (e.g., men or women). Targeting helps get closer to the national population benchmarks in the sample, but it might also increase the cost (Zhang et al. 2020; Neundorf and Öztürk 2023).

9 All respondents who answered the demographic questions are included in this analysis. However, we could not make incentive payments to respondents who did not provide their contact information or left the survey before that stage. This is why the average cost of one participant’s recruitment through incentive-based advertisements can be lower than the expected gain of a social media user from participation in our incentive-based surveys.

10
Supplementary Material sections 3–5 include a list of demographic and political characteristics of samples created with each of the twenty-three advertisements.

11 In Study 1 and Study 3, we measured partisanship by asking respondents if they felt very close, a little close, or not at all close to any political party. Table 4 shows the proportion of respondents in these studies who defined their feelings toward a political party as “very close.” In Study 2, we asked about the propensity to vote rather than partisanship. Table 4 shows the percentage of Spanish respondents that chose 9/10 and 10/10 in this question for any of the parties.

12 The substantive research project on support for democracy, from which our data for Study 2 came, did not include a recontact component. This is why we do not have recontact data for Study 2.

13 The SMS message read, “A short time ago, you filled out a survey with us during the last week. Would you be willing to take a short follow-up survey?”

14 We have also tested a specific type of variation within the neutral category: using human photos versus using images of microphones. The latter was more successful. We present this result in Supplementary Material section 7.

15 We thank the editors for pointing this out.

16 Please see our discussion in Supplementary Material section 1 about how to best navigate Meta’s new policies on political advertisements and prevent spam and bot accounts on Meta.

17 You can access our network at https://www.gla.ac.uk/research/az/democracyresearch/dataandmethods/socialmediaasaresearchtool/.
