# Supplementary material for: Advertising Online Surveys on Social Media: How Your Advertisements Affect Your Study
Source: Public Opin Q. 2025 Jun 16;89(2):335–60. doi: 10.1093/poq/nfaf018 (PMC12369937; doi:10.1093/poq/nfaf018)
Supplement: nfaf018_Supplementary_Data [file nfaf018_supplementary_data.pdf]

Advertising Online Surveys on Social Media: How Your Advertisements Affect Your Study

Supplementary Material

Anja Neundorf (University of Glasgow)

Aykut Öztürk (University of Glasgow)*

Contents

1 Navigating new Meta policies about advertisements **3**

2 Question wordings for surveys **5**

3 Study 1 **7**
- 3.1 Cost Comparisons . . . 7
- 3.2 Sample Characteristics . . . 8
- 3.3 Response quality . . . 10
- 3.4 Advertisements . . . 12
  - 3.4.1 Incentive-based advertisements . . . 12
  - 3.4.2 Neutral advertisements . . . 14
  - 3.4.3 Thematic advertisements . . . 15
  - 3.4.4 Mixed advertisements . . . 16

4 Study 2 **17**
- 4.1 Cost Comparison . . . 17
- 4.2 Sample Characteristics . . . 18
- 4.3 Response Quality . . . 19
- 4.4 Advertisements . . . 20
  - 4.4.1 Incentive-based advertisements . . . 20
  - 4.4.2 Neutral advertisement . . . 21
  - 4.4.3 Thematic advertisements . . . 22

5 Study 3 **23**
- 5.1 Cost comparison . . . 23

*Contact e-mail for any questions related to the article: aykut.ozturk@glasgow.ac.uk

5.2 Sample Characteristics 24
5.3 Response Quality 25
5.4 Advertisements 26
5.4.1 Incentive-based advertisements 26
5.4.2 Neutral advertisement 28
5.4.3 Thematic advertisements 29

6 Statistical Tables for the Response Quality Analysis in the Main Paper 30

7 Neutral Advertisements: Can Humans in Photos Increase their Effectiveness? 31

8 Feasibility of incentive-based advertisements in comparative perspective 32
8.1 Example advertisements 34

1 Navigating new Meta policies about advertisements

Meta has recently implemented new policies that make advertising on the platform more tricky for political scientists. It is important to be aware of these policies to have a smoother advertising experience on the platform.

In an attempt to prevent new scandals like Cambridge Analytica, Meta has developed stricter rules about political advertisements. Most importantly, advertisements with a clear political theme, such as advertisements mentioning political leaders or elections, require the authorization of the advertiser to publish advertisements in that country (Meta, 2023a). If a researcher attempts to publish such an advertisement without authorization, the advertisement is likely to be banned.

This policy does not mean that running a non-political advertisement campaign for a political survey requires special permission, as the survey itself is hosted on a different platform, such as Qualtrics. It also does not mean political scientists can no longer run political/thematic advertisements. It rather means that political scientists should be more careful when designing their advertisements. A thematic/political advertisement, such as the image of a political leader, can only be published by an account authorized to run political advertisements in that country. Securing authorization may not be a problem for researchers running advertisements in a single country, but it will likely be difficult for comparative researchers. On the other hand, some forms of thematic advertisements may not require authorization. For example, our advertisement campaigns in Spain used images of Spanish people holding Spanish flags with an advertisement text saying that the survey was about "current issues." Meta algorithms did not detect any problems with this design.

Secondly, Meta has recently developed new and aggressive algorithms to detect fraudulent or clickbait advertisements (Meta, 2023b, 2023c). These algorithms may flag advertisements by academic researchers as spam. For example, if a researcher creates a new advertisement account and immediately starts spending significant amounts of money, Meta algorithms can take this as an indication of fraudulent advertising. Publishing advertisements during travels or on a computer in which location settings are changed through VPN can lead to a similar result. Some incentive-based advertisements can be flagged as "get rich quick" schemes. To avoid these situations, researchers should invest some time in developing genuine pages on Facebook and Instagram and interacting with their followers. These steps are also commendable in terms of research ethics. For an example of these good practices, you can watch the presentation by Soehl et al. (2023). A researcher may also be able to use the Facebook page of the institution they are employed at for advertisement purposes. This will certainly be helpful in avoiding these issues. Researchers should also design their advertisements more carefully, trying to give more information about what is offered to social media users. For example, the images we design for our most recent incentive-based advertisements are especially clear about who is behind these advertisements by including a logo of our institution.

While these policies have created some new challenges for researchers, Meta has also taken some steps that can help researchers deal with them. Meta has a Research Partnerships Team that supports academic researchers using Meta tools. Academic researchers

having problems with their advertisements can reach this team through an online form (Zoorob, 2023). In our experience, this team does an excellent job of helping researchers, especially when Meta algorithms wrongfully flag an advertisement as spam or fraud. Meta has also started to provide personalized assistence for advertisers having trouble with Facebook advertisement tools, such as setting up the conversion objective in a correct way and provides additional resources teaching how to set up advertisements on the platform.

2 Question wordings for surveys

- How old are you?

  Choose from 18-99.

- What is the highest education level you have attained or are about to attain?

  Does not have a primary school degree
  Primary school degree
  Secondary school degree
  University graduate (including masters and PhD)
  A degree not included here
  Do not know

- What is your gender?

  Male
  Female
  Other & Do not know

- How interested would you say you are in politics?

  Very interested
  Somewhat interested
  Not very interested
  Not at all interested

- Do you usually think of yourself as close to any particular party? [ONLY ASKED IN TURKEY]

  Yes
  No

- Which political party do you feel close to? [ONLY ASKED IN TURKEY]

  Justice and Development Party
  Republican People's Party
  Nationalist Action Party
  Peoples' Democratic Party
  Other [Please write]

- How close do you feel to this political party? [ONLY ASKED IN TURKEY]

  Very close
  Somewhat close
  Not very close

- How likely are you to vote for the following political parties? Can you please show us on a scale from 0 to 10? [ONLY ASKED IN SPAIN]

SUBSTANTIVE QUESTIONS ABOUT DEMOCRACY, INCLUDING EXPERIMENTAL TREATMENTS

- Please choose ‘Do not know’ as your answer to this question.

  (This question was asked at the end of a matrix formed of four questions.)

- Could you please tell us what “democracy” means to you?

- Finally, can you please share your contact information with us? Please enter your phone number and email address below.

3 Study 1

3.1 Cost Comparisons

Table A.1: Cost comparisons in Study 1 in Turkey

Advertisement Strategy		Advertisement Components				Results		Ad-ID
Row		*Incentive*	*Chance of winning*	*Image Type*	*Text Type*	**Days until 250 part.**	**Average Cost**	
1	**Thematic:**	No		Political	Political	2 days	$0.37	12
2	**Incentive:**	Voucher	1 in 7	Incentive	Non-political	2 days	$0.62	5
3	**Incentive:**	Voucher	1 in 1	Incentive	Non-political	2 days	$0.68	4
4	**Mixed:**	Voucher	1 in 1	Incentive	Political	2 days	$0.69	8
5	**Mixed:**	Voucher	1 in 7	Incentive	Political	2 days	$0.69	9
6	**Thematic:**	No		Microphone	Political	6 days	$1.04	7
7	**Neutral:**	No		Microphone	Non-political	7 days	$1.25	1
8	**Neutral:**	No		Male	Non-political	8 days	$1.66	2
9	**Neutral:**	No		Female	Non-political	9 days	$1.69	3
10	**Incentive:**	iPad	1 in 500	Incentive	Non-political	5 days	$1.72	6
11	**Mixed:**	iPad	1 in 500	Incentive	Political	7 days	$2.1	10

Notes:

1. Campaigns are ordered based on the average cost per survey participant.

2. To ensure the comparability between advertisements, we only included the first 250 respondents recruited through each advertisement for the cost analysis. This corresponds to between 11% to 22% of overall samples. See Total N on the next page for an exact comparison.

3. Costs are converted from GBP into USD at the average exchange rate of May 2021 (£1 GBP was equal to $1.4077).

4. In each of the advertisement campaigns, we used targeting based on age, gender, education, and political characteristics.

5. The Ad-ID is the identification number we gave to the advertisement to distinguish the advertisements across the project; this number can be used in reference to the dataset as well.

3.2 Sample Characteristics

In Table A.2, advertisement campaigns are compared with respect to education, gender, and age. The list is ordered based on the proportion of university graduates in each sample since recruiting non-educated respondents is one of the biggest challenges of online recruitment.

Table A.2: Demographic comparisons across samples in Study 1

**Advertisement Category**	**Advertisement Components**			**Results**						**Ad-ID**
	Incentives	Image	Text	University Graduate	Female	Young (18-34)	Old (55+)	Total N	Total Days	
Population of Regime Voters				*12%*	*52%*	*34%*	*28%*			
**Incentive:**	Lottery for voucher	Incentive	Non-political	9%	53%	35%	19%	1,823	4	5
**Incentive:**	Payment to all	Incentive	Non-political	14%	45%	47%	17%	2,138	4	4
**Mixed:**	Lottery for voucher	Incentive	Political	14%	48%	41%	16%	1,370	3	9
**Mixed:**	Payment to all	Incentive	Political	16%	47%	43%	17%	1,806	4	8
**Incentive:**	Lottery for iPad	Incentive	Non-political	17%	41%	54%	11%	1,386	5	6
**Mixed:**	Lottery for iPad	Incentive	Political	19%	43%	53%	9%	1,202	7	10
**Neutral:**	No	Microphone	Non-political	22%	34%	31%	19%	1,349	7	1
**Neutral:**	No	Female	Non-political	22%	35%	26%	29%	1,200	9	3
**Neutral:**	No	Male	Non-political	22%	36%	24%	28%	1,252	9	2
**Thematic:**	No	Political	Political	24%	22%	27%	24%	1,307	3	12
**Thematic:**	No	Microphone	Political	33%	26%	35%	20%	1,134	6	7

Note: Campaigns are ordered based on the proportion of university graduates. The Ad-ID is a number we generated to distinguish the advertisements across the study; this number can be used in reference to the dataset. Total N shows the overall number of respondents recruited with this advertisement. Total Days shows how many days we kept the advertisements open to reach this number.

In Table A.3, advertisements are listed based on the two political characteristics of respondents: political interest and partisanship.

Table A.3: Political Comparisons across Samples

**Advertisement Category**	**Advertisement Components**			**Results**			**Ad-ID**
	Incentives	Image	Text	Very Interested	Partisan	Strong Partisan	
Population of Regime Voters				*10%*	*82%*	*33%*	
**Incentive-based**	Lottery for iPad	Incentive	Non-political	15%	69%	53%	6
**Incentive-based**	Payment to all	Incentive	Non-political	16%	74%	58%	4
**Incentive-based**	Lottery for voucher	Incentive	Non-political	17%	70%	54%	5
**Mixed**	Lottery for voucher	Incentive	Political	18%	86%	49%	9
**Mixed**	Lottery for iPad	Incentive	Political	20%	74%	52%	10
**Mixed**	Payment to all	Incentive	Political	21%	73%	61%	8
**Neutral**	No	Female	Non-political	33%	86%	63%	3
**Neutral**	No	Male	Non-political	35%	86%	69%	2
**Neutral**	No	Microphone	Non-political	37%	83%	68%	1
**Thematic**	No	Microphone	Political	50%	87%	71%	7
**Thematic**	No	Political	Political	53%	85%	69%	12

3.3 Response quality

In Table A.4, we present the comparison of response quality across samples recruited through different advertisements. We have analyzed the impact of advertisement content on response quality by relying on four different outcome variables: passing an attention check, filling a follow-up survey sent to participants one month after the first survey, responding to an open-ended question about the meaning of democracy, and the word count in responses to this open-ended question.

Table A.4: Comparison of Response Quality in Study 1

Advertisement Category	Advertisement Components			Results				Ad-ID
	Incentives	Image	Text	Attention Check (%)	OE: Response (%)	OE: Words Count	Recontact Success (%)	
Incentive-Based	Lower prize (voucher)	Incentive	Non-political	79	84	6	12	5
Incentive-Based	Lower chance of winning (iPad)	Incentive	Non-political	79	86	7	16	6
Mixed	Lower chance of winning (iPad)	Incentive	Non-political	77	87	7	14	10
Mixed	Lower prize (voucher)	Incentive	Non-political	76	89	6	13	9
Mixed	Payment to all	Incentive	Non-political	74	88	6	25	8
Thematic	None	Microphone	Political	74	89	9	4	7
Total				72	87	8	12	
Neutral	None	Female	Non-political	71	83	8	1	3
Neutral	None	Matching images	Egotistic	71	84	8	9	11
Incentive-Based	Payment to all	Incentive	Non-political	71	81	5	16	4
Neutral	None	Microphone	Non-political	70	88	9	7	1
Thematic	None	Political	Political	68	90	9	4	12
Neutral	None	Male	Non-political	67	88	8	5	2

Given that the demographic and political composition of the sample can also affect response quality, we conducted regression analyses, in which we controlled for a series of demographic and political variables. Taking neutral advertisements as reference points, Table A.5 shows how incentive-based, mixed, and thematic advertisements affect response quality. More detailed explanations of the outcome variables are present in the results section of the main document.

Table A.5: Response Quality Analysis for Study 1

	(1)	(2)	(3)	(4)
	Attentive *Logit*	Responded open-ended q. *Logit*	Responded follow-up *Logit*	Word count (sqr) *OLS*
Advertisement types (Neutral ads base category)				
Incentive-based Ads	0.305	0.024	1.106	-0.350
	(0.017)	(0.877)	(0.000)	(0.000)
Thematic Ads	0.090	0.115	-0.338	0.087
	(0.351)	(0.403)	(0.035)	(0.131)
Mixed Ads	0.333	0.502	1.225	-0.181
	(0.009)	(0.005)	(0.000)	(0.013)
Other variables				
College graduate	0.394	0.174	0.079	0.198
	(0.000)	(0.219)	(0.564)	(0.001)
Female	-0.009	-0.459	-0.424	-0.116
	(0.918)	(0.000)	(0.001)	(0.022)
Young	0.512	-0.326	-0.128	0.117
	(0.000)	(0.012)	(0.318)	(0.046)
Old	0.102	0.229	-0.307	0.056
	(0.278)	(0.100)	(0.024)	(0.313)
Political Interest	-0.149	0.251	0.215	0.179
	(0.025)	(0.002)	(0.014)	(0.000)
Strong Partisan	-0.291	0.031	-0.060	-0.050
	(0.001)	(0.789)	(0.606)	(0.316)
Observations	3138	3602	3602	3602

p-values from two tailed tests in parentheses. In the fourth model, we used the square root of the word count variable. All other variables are binary variables.

3.4 Advertisements

3.4.1 Incentive-based advertisements

Incentive-based advertisements in Study 1 can be seen in Figure A.1.

Figure A.1: Advertisements with incentives in Study 1

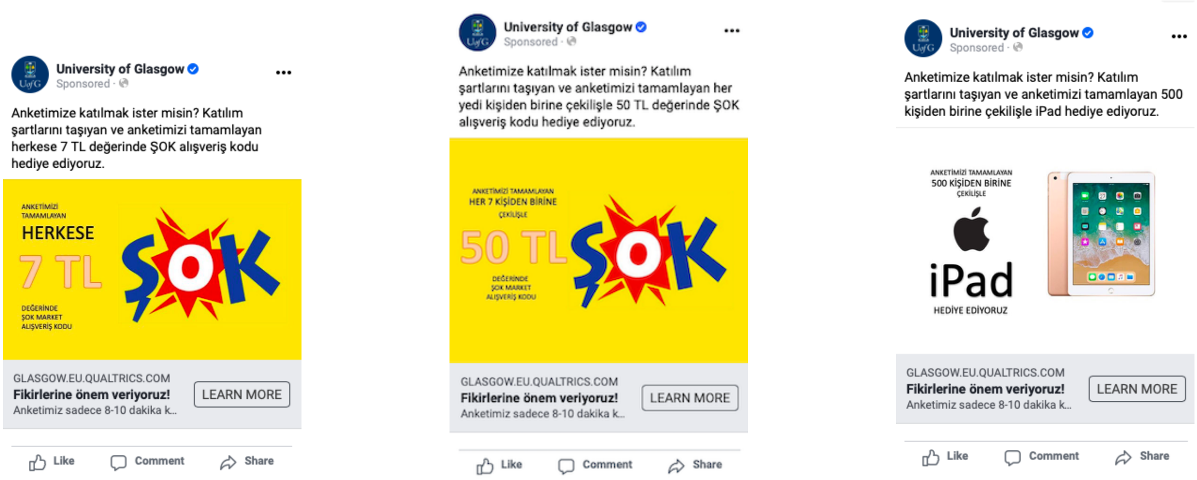

On the left is the advertisement offering a payment to all respondents who successfully completed the survey (winning chance equals one in one). In this advertisement, the advertisement text above the image reads: *"Do you want to participate in our survey? We offer everyone who meets the condition of participation and completes our survey a SOK shopping voucher worth 7 TL."* The advertisement image includes the logo of SOK, which is one of the most widespread grocery store chains in Turkey. The text within the advertisement image reads *"To everyone who completes our survey, a SOK shopping voucher worth 7 TL."*

In the middle is the advertisement with 1 in 7 chance of winning. In this advertisement, the advertisement text above the image reads: *''Do you want to participate in our survey? Through a raffle, we offer one of every seven participants, who meets the condition of participation and completes our survey, a SOK shopping voucher worth 50 TL."* The advertisement image includes the logo of SOK, which is one of the most widespread grocery store chains in Turkey. The text within the advertisement image reads *"To one of every seven people who completes our survey, through a raffle, a SOK shopping voucher worth 50 TL."*

On the right is the advertisement with a 1 in 500 chance of winning, with the promise of an iPad to the winner of the lottery. In this advertisement, the advertisement text above the image reads: *'"Do you want to participate in our survey? Through a raffle, we offer one of five hundred participants who meet the condition of participation and complete our survey an iPad."* The advertisement image includes the image of an iPad. The text within the advertisement image reads *"To one of the five hundred people who completes our survey, through a raffle, an iPad."*

Across all three advertisements, the text below the image reads: *"We value your opinions! Our survey will only take 8-10 minutes."*

3.4.2 Neutral advertisements

Neutral advertisements in Study 1 can be seen in Table A.2.

Figure A.2: Neutral advertisements in Study 1

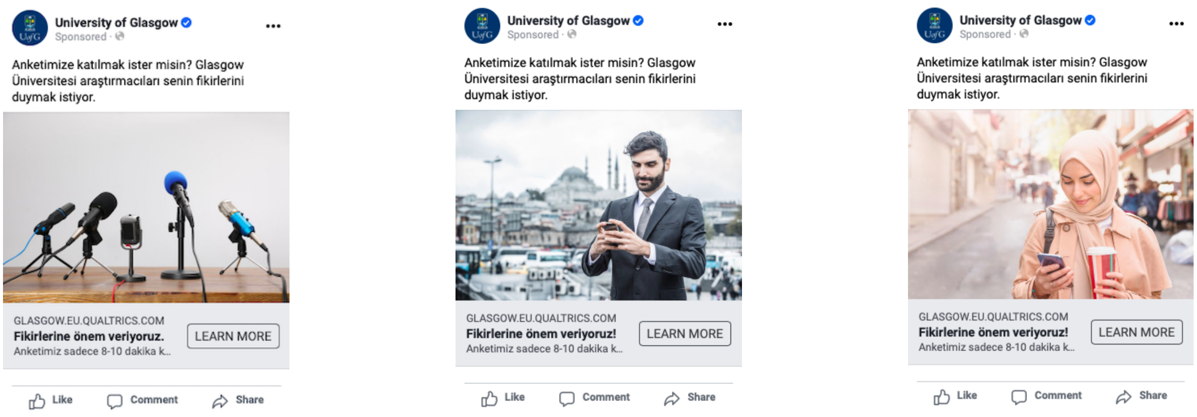

On the left is the baseline neutral advertisement with the image of a microphone. In the middle is a neutral advertisement with the image of a man. On the right is a neutral advertisement with the image of a woman.

Across all these advertisements, the text above the image reads: *"Do you want to participate in our advertisement? University of Glasgow researchers want to hear your opinions."*

Across all these advertisements, the text below the image reads: *"We value your opinions! Our survey will only take 8-10 minutes."*

In the fourth neutral advertisement, we showed the male advertisement to only male social media users and the female advertisement image to only female social media users.

3.4.3 Thematic advertisements

Thematic advertisements in Study 1 can be seen in Figure A.3.

Figure A.3: Thematic advertisements

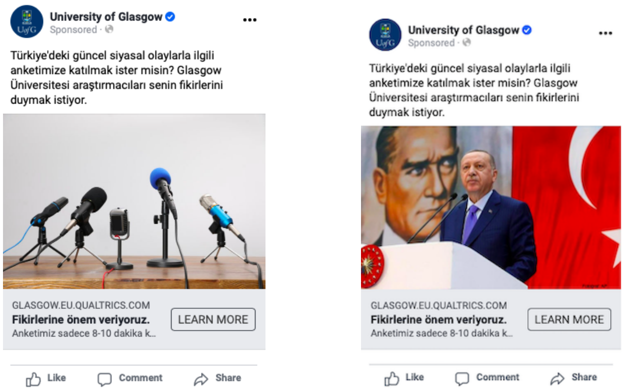

On the left is the thematic advertisement combining the image of a microphone with an advertisement text mentioning the political content of the advertisement. The advertisement text reads *"Do you want to participate in our advertisement on the current political events in Turkey? University of Glasgow researchers want to hear your opinions."*.

On the right is the thematic advertisement combining a political image with the same text mentioning the political content of the survey. In the image, Turkey's authoritarian president Erdogan is giving a speech in front of a picture of Ataturk, Turkey's founding father, and a Turkish flag.

In both advertisements, the text below the image reads: *"We value your opinions! Our survey will only take 8-10 minutes."*

3.4.4 Mixed advertisements

Mixed advertisements in Study 1 can be seen in Figure A.4.

Figure A.4: Mixed advertisements

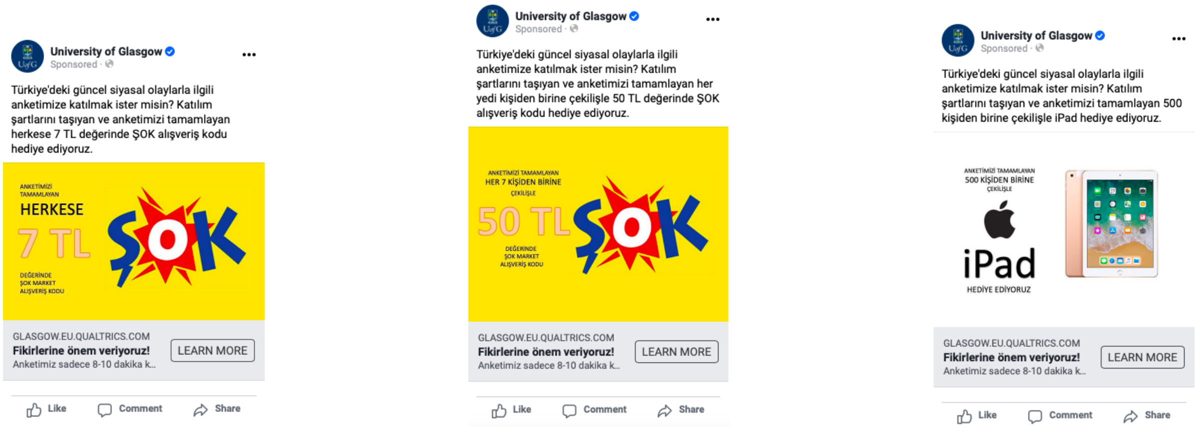

We used three mixed advertisements. These advertisements offer the same incentives as incentive-based advertisements. On the left, the advertisement offers a grocery store voucher worth 7 TL to every participant meeting the conditions of participation and completing the survey. In the middle, the advertisement offers a grocery store voucher worth 50 TL to every one of the seven participants meeting the conditions of participation and completing the survey. To the right, the advertisement offers an iPad, through a lottery, to one of the 500 participants meeting the conditions of participation and completing the survey.

Across all of these advertisements, the advertisement text above the image mentions the political nature of the survey: *"Do you want to participate in our advertisement on the current political events in Turkey?"*. In the second sentence, the advertisement texts mention the incentives that each of the advertisements offer. The second sentence is the same as the sentences used in the incentive-based advertisement above the text.

Advertisement images are also the same as the advertisement images used in incentive-based advertisements.

Across all three advertisements, the text below the image reads: *"We value your opinions! Our survey will only take 8-10 minutes."*

4 Study 2

4.1 Cost Comparison

Table A.6: Comparison of costs across samples in Spain

**Advertisement Strategy**		**Advertisement Components**				**Results**		**Ad-ID**
Row		*Incentive*	*Chance of winning*	*Image Type*	*Text Type*	**Days until 250 part.**	**Average Cost**	
1	**Thematic:**	No		Political	Political	2 days	\$0.16	16
2	**Neutral:**	No		Microphones	Non-political	2 days	\$0.76	15
3	**Incentive:**	Voucher (No brand)	1 in 14	Incentive	Non-political	2 days	\$1.89	13
4	**Incentive:**	Voucher (No brand)	1 in 150	Incentive	Non-political	2 days	\$1.95	14

Notes:

1. Campaigns are ordered based on the average cost per survey participant.

2. To ensure the comparability between advertisements for the cost analysis, we only included the first 250 respondents recruited through each advertisement in this analysis. This corresponds to between 7% to 84% of the overall sample from an advertisement. See Total N on the next page for an exact comparison.

3. Costs are converted from GBP into USD at the average exchange rate of February 2022 (£1 GBP being equal to \$1.3533).

4. In each of the advertisement campaigns, we used targeting based on age, gender, and education.

5. The Ad-ID is the identification number we gave to the advertisement to distinguish the advertisements across the project; this number can be used in reference to the dataset as well.

4.2 Sample Characteristics

We present the demographic and political composition of each sample recruited in Study 2 in Table A.7. Our analysis shows similar patterns to the sample composition in Study 1. Most importantly, incentive-based advertisement campaigns recruited a higher number of non-college-educated people and a higher proportion of people less interested in politics. However, the differences between advertisement campaigns are less stark in Spain, and female respondents were over-represented in incentive-based samples.

Table A.7: Demographic and Political Comparisons in Spain

**Ad. Category**	**Advertisement Components**			**Demographic Results**				**Political Results**	**Total N**	**Total Days**	**Ad ID**
	Incentives	Image	Text	Grad.	Female	Young (18-34)	Old (55+)	Very Interested			
Population				*26%*	*51%*	*22%*	*39%*	*13%*			
**Incentive:**	Lower Prize (Higher Chance)	Incentive	Non-political	36%	59%	22%	43%	17%	304	11	13
**Incentive:**	Lower Chance (Higher Prize)	Incentive	Non-political	46%	62%	26%	38%	19%	438	14	14
**Thematic:**	No	Political	Political	50%	36%	28%	39%	45%	3578	13	16
**Neutral:**	No	Microphone	Non-political	60%	44%	13%	60%	42%	296	11	15

Note: Campaigns are ordered based on the proportion of university graduates. Total N shows the overall number of respondents recruited with this advertisement. Total Days shows how many days we kept the advertisements open to reach this number. The Ad-ID is the identification number we gave to the advertisement to distinguish the advertisements across the project; this number can be used in reference to the dataset as well.

4.3 Response Quality

Secondly, we present a comparison of response quality across samples of Study 2 in Table A.8. Detailed explanations of the outcome variables are present in the results section of the main document.

Table A.8: Response Quality Analysis for Study 2

	(1)	(2)	(3)
	Attentive	Responded to Open Ended	Word count (square root)
Advertisement types (Neutral ads base category)			
Incentive-based Ads	-0.203	1.034	-0.194
	(0.306)	(0.007)	(0.203)
Thematic Ads	-0.124	-0.324	-0.058
	(0.459)	(0.246)	(0.648)
Other variables			
College graduate	0.376	-0.057	0.024
	(0.000)	(0.631)	(0.697)
Female	-0.014	0.056	0.267
	(0.864)	(0.655)	(0.000)
Young	0.318	-0.266	0.249
	(0.003)	(0.053)	(0.001)
Old	-0.413	0.548	0.048
	(0.000)	(0.000)	(0.503)
Political Interest	0.108	0.264	0.203
	(0.069)	(0.003)	(0.000)
Strong Partisan	-0.029	0.041	-0.103
	(0.723)	(0.734)	(0.095)
Observations	3013	3284	3284

p-values from two tailed tests in parentheses. In the fourth model, we used the square root of the word count variable. All other variables are binary variables.

4.4 Advertisements

4.4.1 Incentive-based advertisements

Incentive-based advertisements in Study 2 can be seen in Figure A.5.

Figure A.5: Advertisements in Study 2

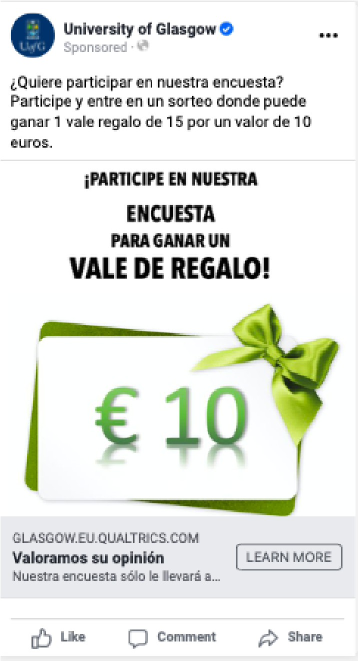

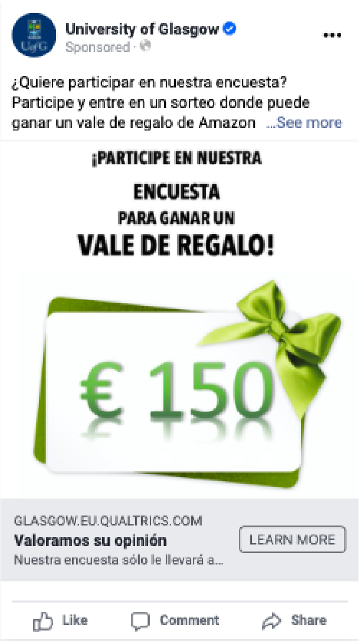

On the left is the incentive-based advertisement with the smaller prize but the bigger chance of winning. Above the image, it reads *"Do you want to participate in our survey? Through a lottery, you can win 1 of 15 vouchers each worth 10 Euros."* Below the advertisement, it reads *"We value your opinion"* and *"Our survey will only take around 10 minutes."*

On the right is the incentive-based advertisement with the bigger prize but the smaller chance of winning. Above the image, it reads *"Do you want to participate in our survey? Through a lottery, you can win a voucher worth 150 Euros."* The advertisement text below the advertisement reads: *"We value your opinions!"* and *"Our survey will only take 10 minutes."*

4.4.2 Neutral advertisement

The neutral advertisement in Study 2 can be seen in Figure A.6.

Figure A.6: Neutral advertisements in Study 2

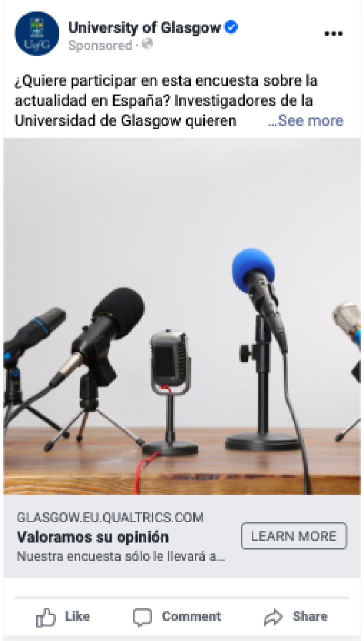

Above the advertisement image, the text reads: *"Do you want to participate in our survey? The University of Glasgow researchers want to hear your opinions."*

The advertisement text below the advertisement reads: *"We value your opinions!"* and *"Our survey will only take 10 minutes."*

4.4.3 Thematic advertisements

The thematic advertisement in Study 2 can be seen in Figure A.7.

Figure A.7: Thematic advertisement in Study 2

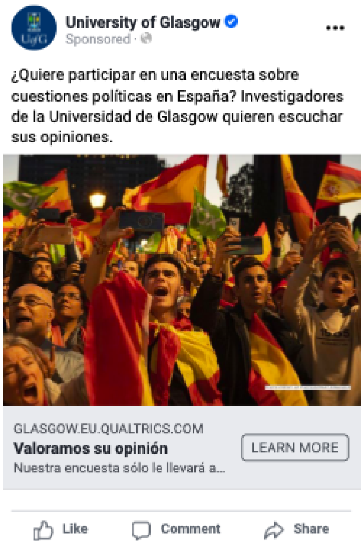

Above the advertisement image, the text reads: *"Do you want to participate in our survey on political issues in Spain? Researchers from the University of Glasgow want to hear your opinions."*

The advertisement text below the advertisement reads: *"We value your opinions!"* and *"Our survey will only take 10 minutes."*

5 Study 3

5.1 Cost comparison

First, we present the comparison of costs across advertisements used in Study 3 in Table A.9.

Table A.9: The comparison of costs across samples in Study 3

	Advertisement Strategy	Incentive Components			Cost	Ad-ID
Row		*Expected Return*	*Chance of Winning*	*Brand Mentioned*		
1	**Thematic**	0	N/a	N/a	$0.10	23
2	**Incentive-Based: Smaller Expected Return**	£0.3	1 in 7	Yes	$0.34	20
3	**Neutral**	0	N/a	N/a	$0.39	22
4	**Incentive-Based: Baseline**	£0.6	1 in 7	Yes	$0.43	18
5	**Incentive-Based: Lower Chance**	£0.6	1 in 14	Yes	$0.48	21
6	**Incentive-Based: Guaranteed Winning**	£0.6	1 in 1	Yes	$0.48	17
7	**Incentive-Based: No brand**	£0.6	1 in 7	No	$0.85	19

Note: Campaigns are listed based on the average cost of participants. Costs are converted from GBP into USD at the average exchange rate of June 2022 (£1 GBP being equal to $1.2318 (Source: https://www.exchangerates.org.uk/GBP-USD-spot-exchange-rates-history-2022.html).) In each of the advertisement campaigns, we used targeting based on age, gender, and education.

5.2 Sample Characteristics

We compare our advertisements used in Study 3 based on the demographic and political composition of the sample in Table A.10. Results are consistent with results from earlier studies. All of the incentive campaigns produce samples that are more representative than thematic and neutral advertisement campaigns. Advertisement campaigns that do not use incentives produce samples that are overly college-educated, male, and old. These samples are also formed of people who are much more interested in politics than average people. An important thing to note is that the sample composition of the neutral campaign is as imbalanced as the sample composition of the thematic campaign.

We do not see significant differences between various types of incentive-based campaigns. No brand advertisement campaign, which was also the least successful in terms of costs, produced the least representative sample as well.

Table A.10: Demographic and Political Sample Compositions in Study 3

**Advertisement Category**	**Advertisement Components**			**Demographic Results**				**Political Results**		**Total N**	**Total Days**	**Ad-ID**
	Incentive type	*Image type*	*Text type*	*Grad.*	*Female*	*Young (18-34)*	*Old (55+)*	*Very Interested*	*Strong Partisan*			
Population				20%	51%	36%	24%	14%	26%			
Incentive	Lower chance	Incentive	Neutral	29	52	37	22	19	36	4.402	9	21
Incentive	Baseline lottery	Incentive	Neutral	30	54	38	23	16	36	4,561	9	18
Incentive	Payment to all	Incentive	Neutral	31	44	54	15	18	39	4,305	10	17
Incentive	Smaller gains	Incentive	Neutral	32	52	36	23	21	37	3,772	10	20
Incentive	No brand	No brand	Neutral	41	48	34	30	29	43	307	2	19
Thematic	None	Protestors	Political	44	23	20	39	54	47	2,536	4	23
Neutral	None	Microphone	Neutral	55	28	17	45	50	39	386	2	22

Note: Campaigns are ordered based on the proportion of university graduates. Total N shows the overall number of respondents recruited with this advertisement. Total Days shows how many days we kept the advertisements open to reach this number. The Ad-ID is the identification number we gave to the advertisement to distinguish the advertisements across the project; this number can be used in reference to the dataset as well.

5.3 Response Quality

Finally, we compare our advertisements based on response quality, as it is presented in Table A.11. Detailed explanations of the outcome variables are present in the results section of the main document.

Table A.11: Response quality analysis for Study 3

	(1)	(2)	(3)	(4)
	Attentive *Logit*	Responded open-ended q. *Logit*	Responded follow-up *Logit*	Word count (sqr) *OLS*
Advertisement types (Neutral ads base category)				
Incentive-based Ads	0.308	0.277	0.259	-0.238
	(0.146)	(0.329)	(0.195)	(0.056)
Thematic Ads	-0.008	0.055	-1.167	0.015
	(0.972)	(0.853)	(0.000)	(0.912)
Other variables				
College graduate	0.304	0.531	0.328	0.280
	(0.000)	(0.000)	(0.000)	(0.000)
Female	0.223	0.003	0.160	0.010
	(0.004)	(0.975)	(0.015)	(0.801)
Young	0.256	-0.094	-0.264	0.187
	(0.003)	(0.343)	(0.000)	(0.000)
Old	-0.041	0.253	-0.222	0.181
	(0.657)	(0.043)	(0.010)	(0.001)
Political Interest	0.058	0.366	0.077	0.252
	(0.214)	(0.000)	(0.062)	(0.000)
Strong Partisan	-0.353	-0.260	0.075	-0.180
	(0.000)	(0.013)	(0.324)	(0.000)
Observations	4930	5322	5322	5322

p-values from two tailed tests in parentheses. In the fourth model, we used the square root of the word count variable. All other variables are binary variables.

5.4 Advertisements

5.4.1 Incentive-based advertisements

Incentive-based advertisements in Study 3 can be seen in Figure A.8 and A.9.

Figure A.8: First group of incentive-based advertisements in Study 3

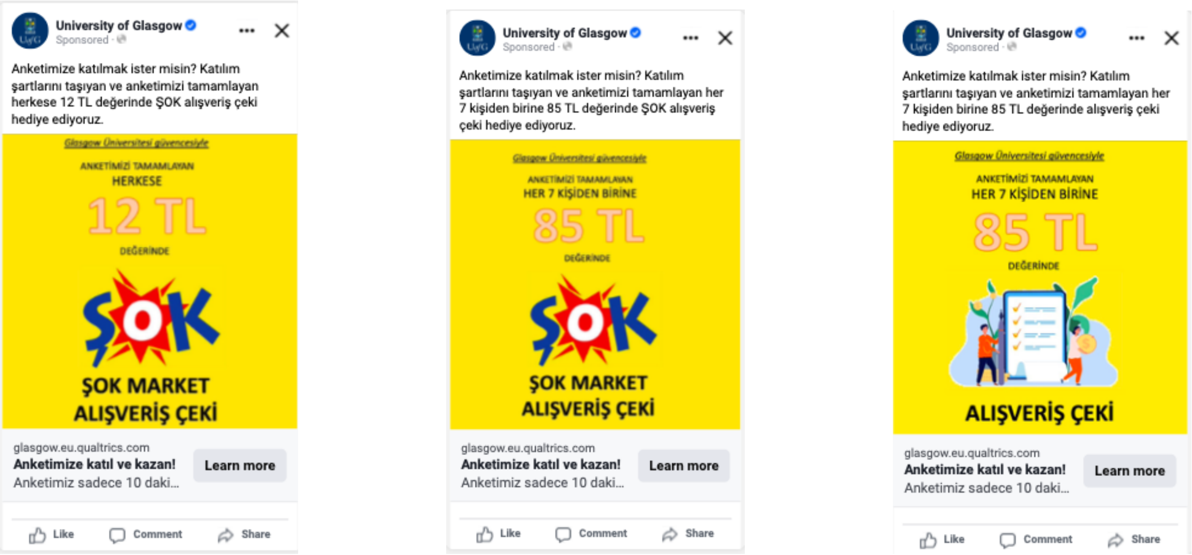

From left to right: guaranteed winning, baseline lottery, lottery with no brands mentioned.

Figure A.9: Second group of incentive-based advertisements in Study 3

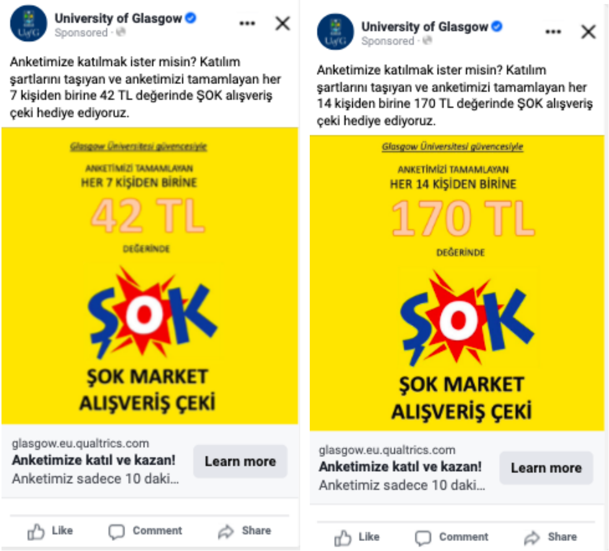

From left to right: Advertisement with the smaller expected return, an advertisement with a very low chance of winning.

Advertisement with guaranteed winning: The text above the image reads *"Do you*

want to participate in our survey? We offer everyone who meets the condition of participation and completes our survey a SOK shopping voucher worth 12 TL." The advertisement image includes a logo of SOK, a supermarket chain in Turkey whose vouchers we delivered. We received permission from SOK to use their logo in our advertisements.

Advertisement with baseline lottery: The text above the image reads *"Do you want to participate in our survey? We offer one of every seven participants who meets the condition of participation and completes our survey a SOK shopping voucher worth 85 TL."* The advertisement image includes a logo of SOK, a supermarket chain in Turkey whose vouchers we are delivering. We received permission from SOK to use their logo in our advertisements. The text within the advertisement image reads: "By the University of Glasgow: A SOK shopping voucher worth 85 TL for every one of seven people who complete our survey."

Advertisement with no brands mentioned: The text above the image reads *"Do you want to participate in our survey? We offer one of every seven participants who meets the condition of participation and completes our survey shopping voucher worth 85 TL."* The advertisement image does not include any logos. The text within the advertisement image reads: "By the University of Glasgow: A shopping voucher worth 85 TL for every one of seven people who complete our survey."

Advertisement with smaller expected return: The text above the image reads *"Do you want to participate in our survey? We offer one of every seven participants who meets the condition of participation and completes our survey a SOK shopping voucher worth 42 TL."* The advertisement image includes a logo of SOK, a supermarket chain in Turkey whose vouchers we are delivering. We received permission from SOK to use their logo in our advertisements. The text within the advertisement image reads: "By the University of Glasgow: A SOK shopping voucher worth 42 TL for every one of seven people who complete our survey."

Advertisement with a very low chance of winning: The text above the image reads *"Do you want to participate in our survey? We offer one of every fourteen participants who meets the condition of participation and completes our survey a SOK shopping voucher worth 170 TL."* The advertisement image includes a logo of SOK, a supermarket chain in Turkey whose vouchers we are delivering. We received permission from SOK to use their logo in our advertisements. The text within the advertisement image reads: "By the University of Glasgow: A SOK shopping voucher worth 170 TL for every one of fourteen people who complete our survey."

Across all advertisements, the advertisement text below the advertisement reads: *"Join our survey and win!"* and *"Our survey will only take 10 minutes."*

5.4.2 Neutral advertisement

The neutral advertisement in Study 3 can be seen in Figure A.10.

Figure A.10: Neutral advertisements in Study 3

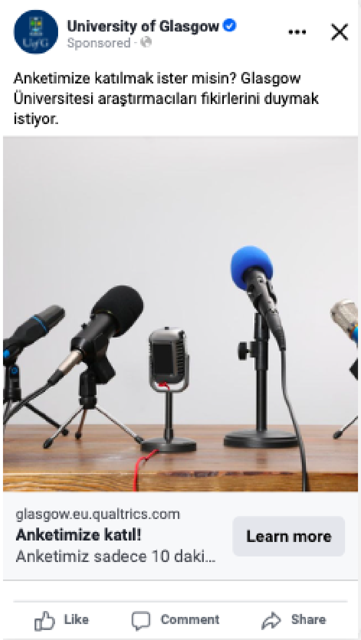

Above the advertisement image, the text reads: *"Do you want to participate in our survey? The University of Glasgow researchers want to hear your opinions."*

The advertisement text below the advertisement reads: *"Join our survey and win!"* and *"Our survey will only take 10 minutes."*

5.4.3 Thematic advertisements

The thematic advertisement in Study 3 can be seen in Figure A.11.

Figure A.11: Thematic advertisement in Study 3

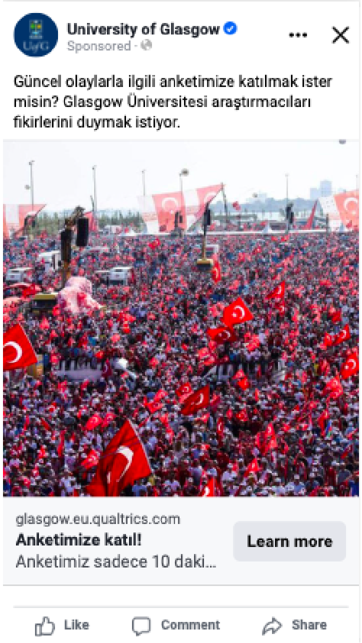

Above the advertisement image, the text reads: *"Do you want to participate in our survey on current issues? The University of Glasgow researchers want to hear your opinions."*

The advertisement text below the advertisement reads: *"Join our survey and win!"* and *"Our survey will only take 10 minutes."*

6 Statistical Tables for the Response Quality Analysis in the Main Paper

Table A.12: Full table for Figure 2 in the main document

	(1)	(2)	(3)	(4)
	Attentive<br>*Logit*	Responded open-ended q.<br>*Logit*	Responded follow-up<br>*Logit*	Word count (sqr)<br>*OLS*
Advertisement types (Neutral ads base category)				
Incentive-based Ads	0.228	0.287	0.894	-0.249
	(0.007)	(0.013)	(0.000)	(0.000)
Thematic Ads	0.067	-0.073	-0.489	0.022
	(0.372)	(0.487)	(0.000)	(0.654)
Mixed Ads	0.325	0.459	1.100	-0.191
	(0.008)	(0.007)	(0.000)	(0.011)
Other variables				
College graduate	0.358	0.270	0.286	0.190
	(0.000)	(0.000)	(0.000)	(0.000)
Female	0.081	-0.111	0.016	0.041
	(0.087)	(0.069)	(0.782)	(0.157)
Young	0.377	-0.223	-0.243	0.173
	(0.000)	(0.001)	(0.000)	(0.000)
Old	-0.127	0.361	-0.249	0.107
	(0.016)	(0.000)	(0.001)	(0.002)
Political Interest	0.024	0.288	0.105	0.222
	(0.457)	(0.000)	(0.005)	(0.000)
Strong Partisan	-0.236	-0.080	0.039	-0.112
	(0.000)	(0.224)	(0.538)	(0.000)
Spain	-0.241	0.103	0.000	0.431
	(0.001)	(0.289)	(.)	(0.000)
Turkey: Study 2	0.237	-0.094	0.537	0.155
	(0.001)	(0.340)	(0.000)	(0.001)
Observations	11081	12208	8924	12208

p-values from two tailed tests in parentheses. In the fourth model, we used the square root of the word count variable. All other variables are binary variables.

7 Neutral Advertisements: Can Humans in Photos Increase their Effectiveness?

Three of our advertisements in Study 1 used neutral advertisements. The baseline neutral advertisement combined a vague text with an image of microphones. In the second and third neutral advertisements, we tested the use of males and females in images combined with neutral text. As we see in Table A.13, using human photos does not help political scientists get cheaper samples; rather, it increases the costs. Starting from Study 2, we, therefore, only used images of microphones for neutral advertisements.

Table A.13: Comparison of neutral advertisements (Study 1: Turkey)

**Summary**	**Content**		**Cost**
	Image	*Text*	
**Neutral**	Microphone	Neutral	\$1.25
**Female Image**	Female	Neutral	\$1.69
**Male Image**	Male	Neutral	\$1.66

We have also experimented with using human photos matching the targeted audience of the advertisement, crossing two age groups (young, old) with two genders (male, female). Thus, for example, old male Facebook users saw a photo of an old man. As photos matched the gender and age of the audience, we used an egotistic text: *"You are selected for this advertisement! University of Glasgow researchers want to hear your opinions."* We did not observe a positive effect of this strategy as well.

8 Feasibility of incentive-based advertisements in comparative perspective

Based on studies in Turkey and Spain, our article concludes that incentive-based advertisements are superior to neutral and thematic advertisements. To what extent are our incentive-based advertisements feasible worldwide?

Between May and June 2023, we ran advertisements in 32 countries worldwide. Our goal was to recruit at least 1,500 respondents in each country. All of our advertisements used the same template of incentive-based advertisements to win one voucher worth $500. Our advertisement image was a voucher from an online shopping website. In many countries, this was Amazon.com or its local branches. In some other countries, we used images from local shopping websites, such as Jumia (in Kenya) and MercadoLibre (in Latin America). If we could not find any local shopping websites, we used the images of Visa Prepaid Debit Cards. We have generally delivered our advertisements targeting gender, age, and education categories separately. It is important to note that these advertisement campaigns were not controlled comparisons, as it was the case with the advertisement campaigns presented in the main text of this article. There were no thematic or neutral advertisements that served as a comparison group.

Results from our advertisement campaigns, in terms of sample composition and cost, are presented in Table A.14. We think that these results show the feasibility of incentive-based advertisement campaigns worldwide. The overall cost per completed response was 1$ or less in 26 countries. Only in three countries the cost was over 3$: Australia, Hong Kong, and Singapore. We were also satisfied with the sample composition, especially in Latin American countries, where the language of our advertisements overlapped with the dominant language among both educated and uneducated people in the country.

Definitions of variables of Table A.14:

- *Female*: % of women
- % of respondents with a *university degree*
- *Young*: % of respondents aged 18-25
- *Old*: % of respondents aged 55 and older
- *Mean age* of the sample
- % of respondents *very interested in politics*
- *Attentive*: % who passed an attention check related to an experimental treatment
- *Open-ended question*: % who responded and average number of words used
- *Number of respondents* who completed the survey
- *Costs per completed survey*, including the prize draw costs of $500 (one-off)

Table A.14: Sample characteristics and costs of comparative public opinion surveys

Country	Female	Uni degree	Young	Old	Mean age	Politically very int.	Attentive	Open ended question		N Compl. survey	Costs per survey (in $)
								Responded	N words		
Argentina	54.7%	12.3%	25.5%	38.7%	47.3	21.4%	83.9%	97.6%	11.7	2,014	0.25
Australia	59.3%	48.5%	50.2%	30.7%	40.6	36.5%	85.5%	97.4%	18.2	1,169	3.15
Bangladesh	36.8%	52.1%	64.4%	10.9%	31.5	11.3%	81.9%	97.4%	19.4	2,107	0.33
Bolivia	44.6%	33.4%	35.8%	26.9%	42.1	17.9%	81.7%	97.3%	13.4	1,586	0.81
Chile	62.7%	13.7%	23.5%	41.1%	48.1	20.4%	82.5%	98.0%	15.0	2,085	0.67
Colombia	52.2%	18.8%	30.0%	38.4%	45.6	21.9%	83.0%	98.1%	15.3	1,705	0.33
Dominican Republic	52.3%	20.3%	54.5%	18.6%	35.6	16.8%	79.1%	97.2%	16.0	1,675	0.45
Ecuador	51.8%	21.3%	41.4%	26.4%	40.1	18.0%	79.5%	98.1%	15.2	1,619	1.21
El Salvador	46.5%	14.2%	43.7%	23.5%	39.9	17.9%	82.5%	97.1%	15.4	1,578	0.64
Ghana	33.7%	57.1%	77.4%	3.9%	29.4	21.0%	86.3%	98.4%	17.4	1,849	0.31
Guatemala	43.8%	11.9%	47.3%	17.1%	37.1	18.1%	80.2%	96.8%	14.5	1,541	0.70
Honduras	49.0%	15.2%	51.5%	16.5%	36.7	13.8%	81.8%	97.7%	15.7	1,590	0.31
Hong Kong	73.7%	55.5%	47.6%	6.6%	35.9	14.2%	79.4%	96.7%	13.6	1,166	3.13
India	39.3%	57.8%	56.7%	19.8%	35.3	18.4%	79.6%	96.9%	15.0	1,865	0.30
Kenya	42.3%	53.5%	70.6%	4.6%	30.7	25.2%	83.3%	98.6%	14.3	2,483	0.15
Malaysia	55.3%	58.5%	66.5%	10.9%	32.4	15.8%	81.9%	97.3%	16.2	1,580	1.01
Mexico	50.1%	22.3%	26.6%	40.0%	47.0	23.5%	83.7%	97.4%	13.9	1,638	0.52
Nicaragua	47.9%	28.2%	42.5%	19.4%	39.1	11.2%	81.1%	97.6%	17.7	1,691	0.81
Nigeria	39.4%	55.1%	68.5%	6.8%	30.5	28.4%	86.4%	99.4%	18.4	2,621	0.21
Pakistan	50.6%	69.7%	51.2%	14.8%	35.9	16.3%	83.3%	97.2%	15.7	3,262	0.19
Paraguay	49.9%	21.2%	42.8%	20.0%	38.9	21.5%	84.2%	97.8%	15.0	1,601	0.51
Peru	51.4%	20.2%	34.1%	32.8%	43.2	22.0%	82.5%	97.7%	15.4	1,567	0.72
Philippines	68.2%	42.0%	37.4%	25.0%	41.2	18.4%	78.7%	97.4%	14.2	1,910	1.09
Singapore	48.2%	49.2%	69.4%	10.2%	31.6	15.6%	79.9%	98.6%	15.2	823	4.19
South Africa	53.1%	25.2%	53.1%	19.3%	36.4	18.0%	82.1%	98.4%	17.2	2,736	0.21
Spain	58.5%	19.6%	34.3%	33.7%	43.6	24.6%	82.3%	96.7%	12.6	1,545	1.94
Tanzania	30.6%	63.4%	70.9%	7.0%	32.0	24.5%	84.8%	98.6%	18.3	1,735	0.53
Uganda	36.2%	53.6%	82.2%	2.2%	28.4	18.5%	83.6%	98.9%	17.1	2,462	0.33
Venezuela	47.2%	35.7%	25.7%	36.3%	46.5	16.4%	86.1%	98.8%	17.2	2,197	0.22
Zambia	45.9%	43.8%	66.0%	8.5%	32.4	25.3%	80.2%	99.3%	16.4	1,956	0.43
United Kingdom	63.6%	57.5%	29.1%	44.4%	48.5	38.1%	85.7%	97.3%	16.1	1,682	0.70
United States	66.0%	40.3%	57.9%	23.7%	36.5	37.1%	84.6%	96.9%	16.3	1,636	0.83
**Average**	50.2%	37.2%	49.3%	21.2%	38.1	20.9%	82.5%	97.8%	15.7	1,834	0.85

8.1 Example advertisements

In all 32 countries, we used variations of the same template. You can see the template and its use in two different contexts in Figure A.12.

Figure A.12: Examples from the cross-national data collection

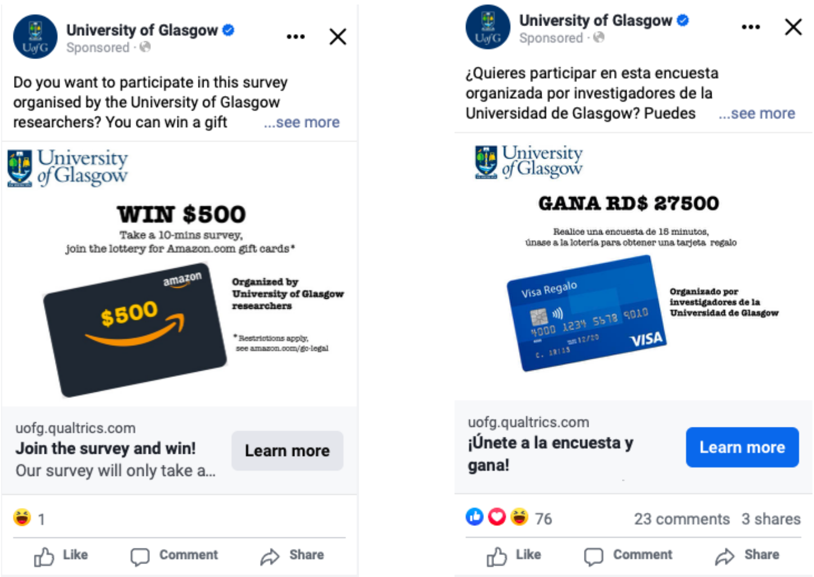

On the left is the advertisement we used in the United States. Following the guidelines Amazon delineates, we used the image of an Amazon gift card in our advertisements.

On the right is the advertisement we used in the Dominican Republic. The text in this advertisement is the translation of the text we used in the English advertisement. Since Amazon does not operate in the United States, we used the image of a Visa gift card.

References

Meta. (2023a). *About ads about social issues, elections or politics*. Retrieved July 17, 2023, from https://www.facebook.com/business/help/167836590566506?id=288762101909005

Meta. (2023b). *Best practices to improve ad quality and performance*. Retrieved July 17, 2023, from https://www.facebook.com/business/help/167836590566506?id=288762101909005

Meta. (2023c). *How to avoid posting clickbait on facebook*. Retrieved July 17, 2023, from https://www.facebook.com/business/help/503640323442584?id=208060977200861

Soehl, T., Chen, Z., & Aaron, E. (2023). *Using targeted social media advertising to sample global migrant populations*. Retrieved July 17, 2023, from https://www.youtube.com/watch?v=_IGGccR8QR4&feature=youtu.be

Zoorob, M. (2023). *Meta ads for academic research: Tips and how to get help*. Retrieved July 17, 2023, from https://www.gla.ac.uk/research/az/democracyresearch/dataandmethods/socialmediaasaresearchtool/webinarseries/#29november2022-15h(gmt)%3Aacademicadvertisingwithmeta%3Atipsandadvice(michaelzoorob%2Cmeta)
